# Mucin Increases the Virulence of Enterotoxigenic *Escherichia coli*

**DOI:** 10.3390/biology15191722

**Published:** 2026-10-01

**Authors:** Sofia Razzaq Meo, Adithi Ravikumar, Christophe Noppe, Patrick De Clercq, Tom Defoirdt

**Affiliations:** 1 Center for Microbial Ecology and Technology (CMET), Department of Biotechnology, Ghent University, Frieda Saeysstraat 1, 9052 Ghent, Belgium; sofiarazzaq.meo@ugent.be (S.R.M.);; 2 Laboratory of Agrozoology, Department of Plants and Crops, Ghent University, 9000 Ghent, Belgium

**Keywords:** mucin, biofilm formation, antivirulence therapy, virulence factors, ETEC

## Abstract

Enterotoxigenic *Escherichia coli* (ETEC) is an important pathogen of humans and other mammals (such as piglets and calves). Mucin, a glycoprotein that forms the major fraction of the intestinal mucus layer, is a key host substance that ETEC encounters in the host environment. This study investigated whether physiologically relevant concentrations of mucin modulate virulence-related phenotypes in ETEC. Growth of ETEC remained unaffected across mucin concentrations ranging from 50 mg/L to 1000 mg/L. Mucin increased swimming motility in a concentration-dependent manner, with motility doubling at the highest concentrations tested relative to the mucin-free control. Further, mucin also increased biofilm formation in a dose-dependent manner, with approximately 4.5-fold higher biofilm levels at the highest concentration. Finally, mucin increased the virulence of ETEC in the invertebrate infection model *Galleria mellonella*, resulting in a 1.5- to 3.3-fold increase in larval mortality caused by mucin-treated ETEC compared with the untreated ETEC group. These findings indicate that ETEC responds to mucin as a host-derived cue, promoting virulence-associated traits linked to colonization and infection. Understanding this host–pathogen interaction may lead to the development of novel control strategies against ETEC, which is an urgent societal challenge in the context of rising antibiotic resistance.

## Simple Summary

Diarrheal disease caused by certain pathotypes of the bacterium *Escherichia coli* is a major health problem worldwide, particularly among young children and travelers in low- and middle-income countries. Before these bacteria can cause illness, they must first pass through the protective mucus layer lining the intestine. This mucus contains a substance called mucin, which may provide bacteria with information about their surroundings and help them adapt to the gut. In this study, we investigated whether mucin changes the behavior of a disease-causing pathotype of *E. coli*. We found that exposure to mucin made the bacteria more mobile and increased their ability to form groups of bacteria attached to surfaces, a behavior that can help them establish and remain in the gut. We also found that bacteria exposed to mucin led to higher mortality in *Galleria mellonella*, an insect commonly used as a model to study bacterial infections. These findings suggest that mucin can influence how these bacteria behave during the early stages of infection. Understanding how bacteria respond to mucin may help researchers develop new approaches that block these responses and reduce bacterial disease without directly killing bacteria, potentially reducing the pressure that drives antibiotic resistance.

## 1. Introduction

Enterotoxigenic *Escherichia coli* (ETEC) is one of the leading causes of watery diarrhea, particularly in low- and middle-income countries (LMICs). Although ETEC is widely recognized as a primary cause of traveler’s diarrhea among individuals visiting endemic regions, it also represents a significant pathogen affecting young children living in these areas. Globally, ETEC is estimated to cause approximately 84.4 million diarrheal episodes each year [1].

ETEC initiates infection by colonizing the small intestinal epithelium through colonization factors, most of which are plasmid-encoded, that mediate bacterial attachment to the host mucosa [2]. This interaction facilitates the delivery of enterotoxins, including heat-labile toxin (LT) and/or heat-stable toxin (ST), which alter intestinal electrolyte transport and promote fluid accumulation within the intestinal lumen, leading to the characteristic watery diarrhea associated with ETEC infection [3]. However, successful colonization requires ETEC to first adapt to the complex environment of the gastrointestinal (GI) tract by sensing and responding to host-derived cues [4].

During the colonization process, ETEC must first traverse the intestinal mucus layer, one of the earliest host barriers encountered in the GI tract. The mucus layer is a dense viscoelastic barrier primarily composed of highly glycosylated mucin glycoproteins [5]. As the major structural constituents of mucus, mucins are essential for maintaining mucosal barrier integrity and protecting the underlying epithelium from microbial invasion [6].

Although the mucus layer restricts pathogen access to epithelial cells, it is not merely a passive physical barrier. Mucin glycoproteins and their associated glycans also serve as nutrient sources, substrates for bacterial attachment, and environmental cues that can influence bacterial physiology and virulence gene expression [7,8,9]. For example, mucin induces the expression of virulence-associated genes in *Campylobacter jejuni*, including genes involved in toxin production, host cell invasion, and mucin degradation [10] and regulates the transition between motile and sessile lifestyles in *Vibrio cholerae* [11]. Mucin has a marked increasing impact on motility (called surfing motility) of several bacteria, including *E. coli* MG1655 and Enterotoxigenic *E. coli* (EHEC) [12]. Further, mucin increased protease activity, swimming motility, biofilm formation, and virulence towards sea bass larvae of *Vibrio anguillarum* [13]. Mucin has similarly been shown to promote biofilm formation in environmental strains of *E. coli* [14]. These studies suggest that enteric pathogens have evolved mechanisms to sense and respond to mucin-derived molecules as cues for adapting to the intestinal environment.

Despite these observations in other enteric pathogens, it remains unknown whether ETEC similarly responds to mucin by modulating virulence-associated traits. In particular, it is unclear whether physiologically relevant mucin concentrations affect ETEC physiology, or whether mucin exposure modulates virulence. Addressing this gap is important not only for understanding the early stages of ETEC infections but also for identifying how ETEC senses and responds to the host, which may reveal targets for anti-virulence interventions that aim to disarm the pathogen by disrupting its virulence-regulatory network rather than killing it. Because anti-virulence strategies do not directly inhibit bacterial viability, they are expected to impose substantially lower selective pressure for resistance development than conventional antibiotics do [15,16], making them an attractive alternative given the rising prevalence of antibiotic-resistant ETEC strains. This work therefore aimed to determine how physiologically relevant concentrations of mucin influence ETEC growth kinetics, motility, and biofilm formation, and to assess whether these mucin-induced responses alter bacterial pathogenicity in vivo.

## 2. Materials and Methods

### 2.1. Bacterial Strain and Culture Conditions

ETEC strain H10407 was used throughout this study. Bacterial stocks were stored at −80 °C in Luria–Bertani (LB) broth supplemented with 20% (*v*/*v*) glycerol. For experiments, bacteria were cultured overnight in LB broth at 37 °C with shaking at 100 rpm from frozen stocks. Cultures were then diluted into fresh LB broth and incubated under the same conditions before downstream assays. Bacterial density was assessed by measuring OD_600_ using a spectrophotometer (Tecan Infinite M200 Pro, Tecan, Mechelen, Belgium).

### 2.2. Mucin Stock Solution

Partially purified porcine gastric mucin (Type III; bound sialic acid content 0.5–1.5%; Sigma-Aldrich, Darmstadt, Germany) was used as the mucin source in this study. A stock solution (2500 mg/L) was prepared by gradually dissolving the mucin in distilled water with gentle heating and stirring and sterilized by autoclaving before use.

### 2.3. Effect of Mucin on Bacterial Growth Kinetics

To assess the impact of mucin on the growth kinetics of ETEC H10407, the mucin stock solution (2500 mg/L) was diluted in distilled water to prepare working solutions of 100, 200, 500, 1000, and 2000 mg/L. These solutions were mixed 1:1 (*v*/*v*) with double-strength LB broth and inoculated with overnight-grown ETEC H10407 cultures to obtain final mucin concentrations of 50, 100, 250, 500, and 1000 mg/L and a starting OD_600_ of 0.01. Sterile LB broth without bacteria was included as a blank control. For each condition, 200 µL of culture suspension was transferred into a transparent 96-well microtiter plate, with three biological replicates per treatment. Plates were incubated at 37 °C, and bacterial growth was monitored by measuring OD_600_ at hourly intervals using a plate reader (Tecan Infinite M200 Pro). OD_600_ measurements were corrected by subtracting the average value obtained from the blank control.

### 2.4. Effect of Mucin on Swimming Motility

Swimming motility of ETEC H10407 was assessed using a soft agar-based assay, as previously described [15], with minor modifications. Briefly, an overnight ETEC culture was diluted to an OD_600_ of 0.05. The mucin stock solution (2500 mg/L) was diluted in distilled water to prepare intermediate concentrations of 100, 200, 500, 1000, and 2000 mg/L. These solutions were mixed 1:1 (*v*/*v*) with double-strength soft LB agar (0.6% agar) to obtain final mucin concentrations of 50, 100, 250, 500, and 1000 mg/L. Double-strength agar mixed with distilled water served as a mucin-free control. Following solidification, 2 µL of bacterial suspension was inoculated onto the center of each plate. Plates were incubated upright at 37 °C for 8 h, after which motility halo diameters were measured and normalized to the mean diameter of mucin-free control plates, which was set at 100%. The assay was performed in two independent experiments, with six replicate plates per mucin concentration in each experiment.

### 2.5. Effect of Mucin on Biofilm Formation

Biofilm formation was quantified using a crystal violet staining-based assay, as previously described [15]. Briefly, the OD_600_ of an overnight ETEC H10407 culture was adjusted to 0.1 in double-strength LB medium. The mucin stock solution was diluted in distilled water to prepare working concentrations of 100, 200, 500, 1000, and 2000 mg/L, which were mixed 1:1 (*v*/*v*) with the diluted bacterial culture to yield final mucin concentrations of 50, 100, 250, 500, and 1000 mg/L. A mucin-free control and a sterile medium blank control were included. Aliquots of 200 µL of each mixture were transferred into a transparent 96-well microtiter plate. Plates were incubated at 37 °C for 8 h. Following incubation, planktonic cell density (OD_600_) was measured, after which the medium was removed, and wells were rinsed three times with phosphate-buffered saline to remove non-adherent cells. Attached biofilms were fixed with methanol for 20 min, air-dried, and stained with 1% crystal violet (Sigma-Aldrich) for 15 min at room temperature. Wells were then rinsed with distilled water to remove residual stain, and bound crystal violet was solubilized in 95% ethanol for 30 min before measuring absorbance at 570 nm. Both OD_600_ and OD_570_ values were blank-corrected, and biofilm was expressed as the OD_570/_OD_600_ ratio to normalize biofilm biomass to planktonic growth. The assay was performed in three independent experiments, with 4 replicate wells per mucin concentration in each experiment.

### 2.6. Effect of Mucin on ETEC Virulence in the Galleria Mellonella Infection Model

Before the challenge test, *Galleria* larvae were reared at the Laboratory of Agrozoology (Ghent University) on an artificial diet consisting of wheat flour, milk powder, beeswax, yeast, honey, glycerol, and sugar [17], and maintained at 25 °C. Challenge tests were performed as described previously, with slight modifications [18]. Briefly, ETEC H10407 cultures were grown in LB medium with or without 1000 mg/L mucin at 37 °C for 24 h with shaking at 100 rpm. Following incubation, bacterial density was determined by measuring OD_600_, and cultures were adjusted to an OD_600_ of approximately 1. Bacterial suspensions were serially diluted in phosphate-buffered saline (PBS), and 10 µL of the diluted suspension was injected into the last left proleg of individual larvae using a microfine insulin syringe with a 30-gauge 8 mm long needle, delivering a final inoculation dose of approximately 10^4^ cells per larva. For the mucin-treated group, mucin was added to the bacterial suspension at a final concentration of 1000 mg/L, prior to injection. A PBS-only injection control and an untreated ETEC-only group (bacteria grown without mucin) were included alongside the mucin-treated group. To assess the potential toxicity of mucin itself, an additional group of larvae was injected with a 1000 mg/L mucin solution in the absence of bacteria. Following infection, larvae were transferred to Petri dishes and incubated at 37 °C in the dark. Twenty larvae were used per treatment group. Larval survival was monitored at 24, 48, 72, 96, and 120 h post-infection. Larvae were considered dead when they displayed complete melanization and/or failed to respond to physical stimulation [19]. Survival data were analyzed using the Kaplan–Meier method. The assay was performed in two independent experiments.

### 2.7. Data Analysis

Statistical analyses were performed using IBM SPSS Statistics (Version 29.0). Swimming motility and biofilm formation data were analyzed using one-way analysis of variance (ANOVA), followed by Tukey’s HSD post hoc test. A *p*-value < 0.05 was considered statistically significant. Survival of *G. mellonella* larvae was analyzed using the Kaplan–Meier method, and survival curves were compared using the log-rank (Mantel–Cox) test. Statistical significance was indicated as * *p* < 0.05, ** *p* < 0.01, *** *p* < 0.001.

## 3. Results

### 3.1. Physiological Concentrations of Mucin Do Not Affect Growth Kinetics of ETEC H10407

**Figure 1:**
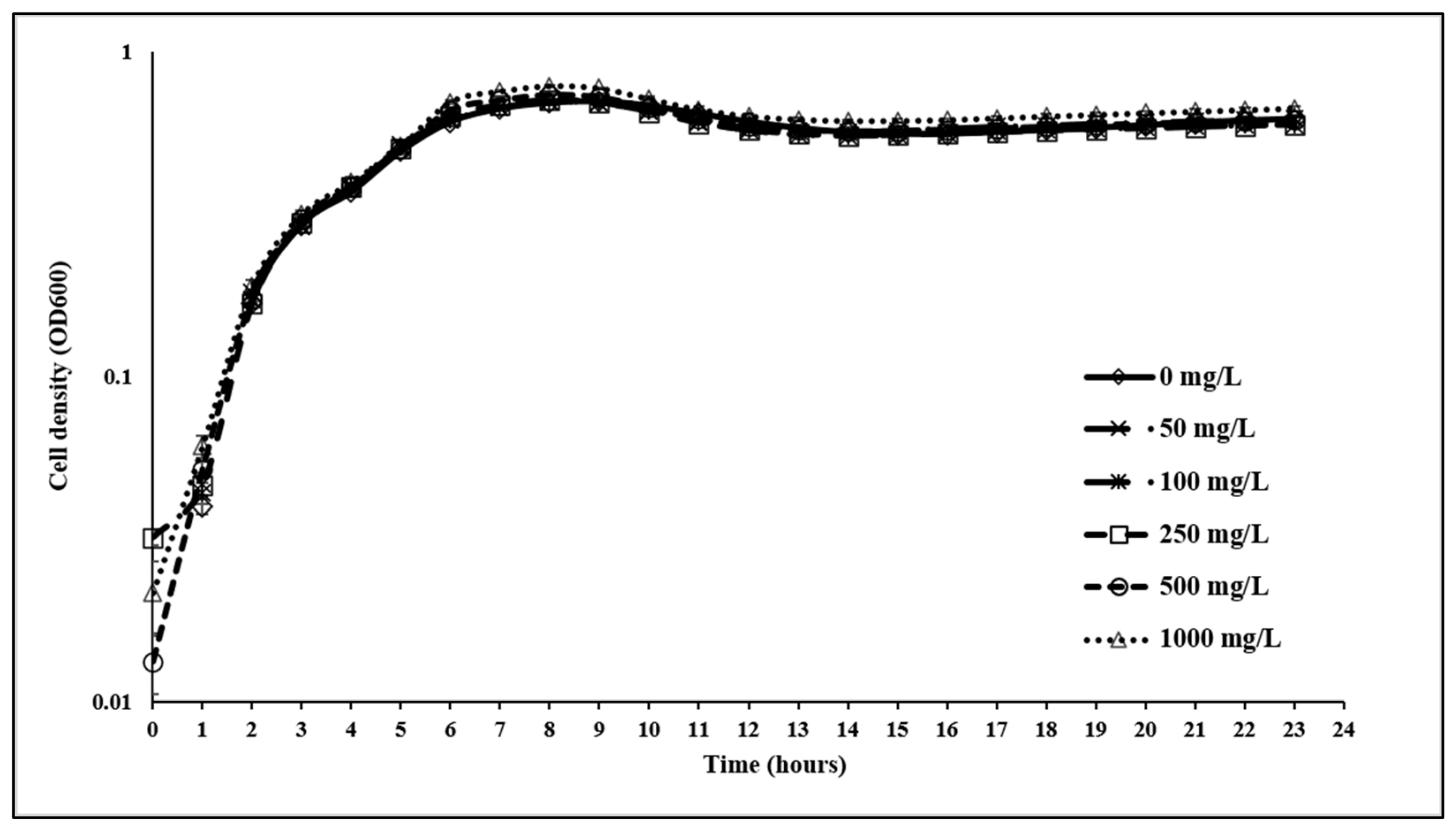
Growth kinetics of ETEC H10407 in the absence and presence of physiologically relevant concentrations of mucin (i.e., 50–1000 mg/L). Optical density at 600 nm (OD_600_) was measured hourly and blank-corrected. Error bars represent the standard deviation of three replicate cultures. The results are representative of two independent experiments.

To determine whether mucin affects the growth kinetics of ETEC H10407, bacterial growth was monitored across a range of physiologically relevant mucin concentrations. This analysis was performed to identify concentrations suitable for use in subsequent virulence-associated assays without confounding effects on bacterial growth. No differences in growth kinetics were observed between mucin-treated cultures and the mucin-free control at any concentration tested (Figure 1), indicating that mucin does not influence the growth of ETEC H10407 under these conditions.

### 3.2. Mucin Increases the Swimming Motility of ETEC H10407

**Figure 2:**
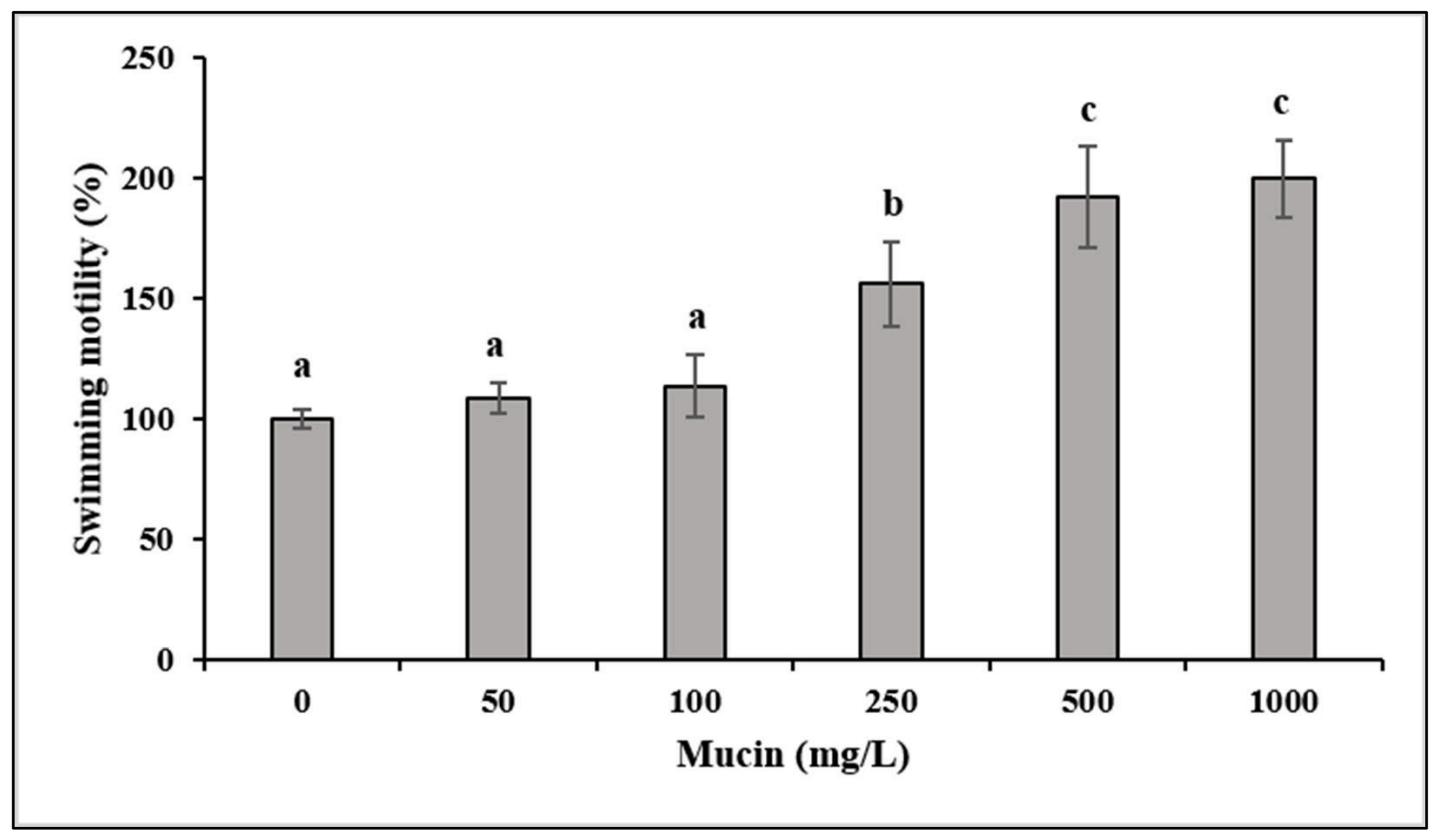
Swimming motility of ETEC H10407 in the absence and presence of mucin on soft LB agar. Motility of the mucin-free control was set at 100% and the other treatments were normalized accordingly. The error bars represent the standard deviation of six replicate plates per treatment. Bars showing different letters are significantly different from each other (one-way ANOVA followed by Tukey’s HSD post hoc test, *p* < 0.05). The results are representative of two independent experiments.

The effect of mucin on flagella-mediated swimming motility of ETEC H10407 was evaluated using a soft-agar motility assay. Initial experiments showed that the inoculum densities used in our previous work (i.e., OD_600_ of 1) resulted in extensive bacterial migration over the agar surface after overnight incubation, especially at the highest mucin concentrations. From 250 mg/L onwards, the motility was so strong that the bacteria had covered the whole plates, limiting the ability to identify differences between treatments. Assay conditions were therefore adjusted (starting OD_600_ of 0.05 and determining motility halos after 8 h of incubation). Under these conditions, mucin significantly affected the swimming motility of ETEC H10407 (Figure 2). Motility at 50 and 100 mg/L mucin did not differ significantly from the mucin-free control, whereas exposure to 250 mg/L mucin significantly increased motility relative to the control, reaching 156% of control levels. Motility increased further at 500 and 1000 mg/L mucin, reaching 192% and 200% of control levels, respectively.

### 3.3. Mucin Increases the Biofilm Formation of ETEC H10407

**Figure 3:**
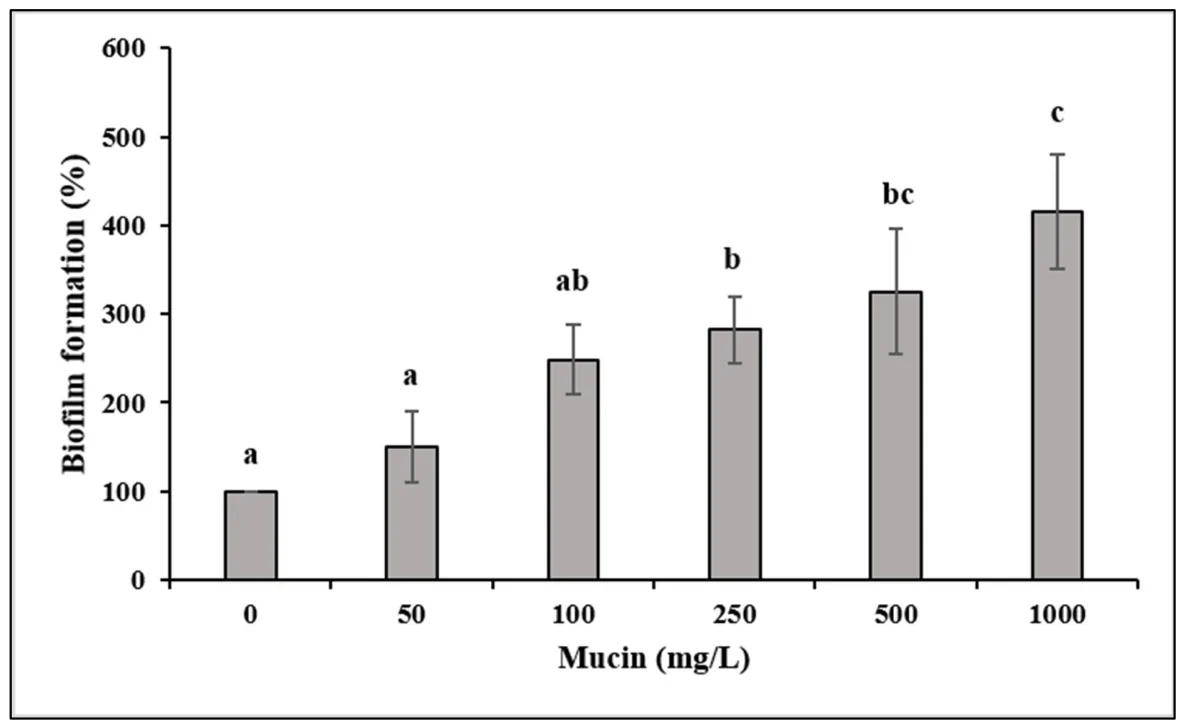
Biofilm formation of ETEC H10407 in the absence and presence of mucin. Biofilm formation was assessed using crystal violet staining. The biofilm formation of untreated ETEC was set at 100%, and the other treatments were normalized accordingly. The error bars show the standard deviation of three independent experiments. Bars showing different letters are significantly different from each other (one-way ANOVA followed by Tukey’s HSD post hoc test, *p* < 0.05).

To determine whether mucin influences biofilm formation by ETEC H10407, a crystal violet-based assay was performed across a range of physiologically relevant mucin concentrations. Across three independent experiments, biofilm biomass increased with increasing mucin concentration (Figure 3), with minimal change observed at the lowest concentration tested (50 mg/L), rising from approximately 2.3-fold at 100 mg/L to 4.5-fold at 1000 mg/L relative to the mucin-free control.

### 3.4. Mucin Increases the Virulence of ETEC in the Invertebrate Model Galleria Mellonella

**Figure 4:**
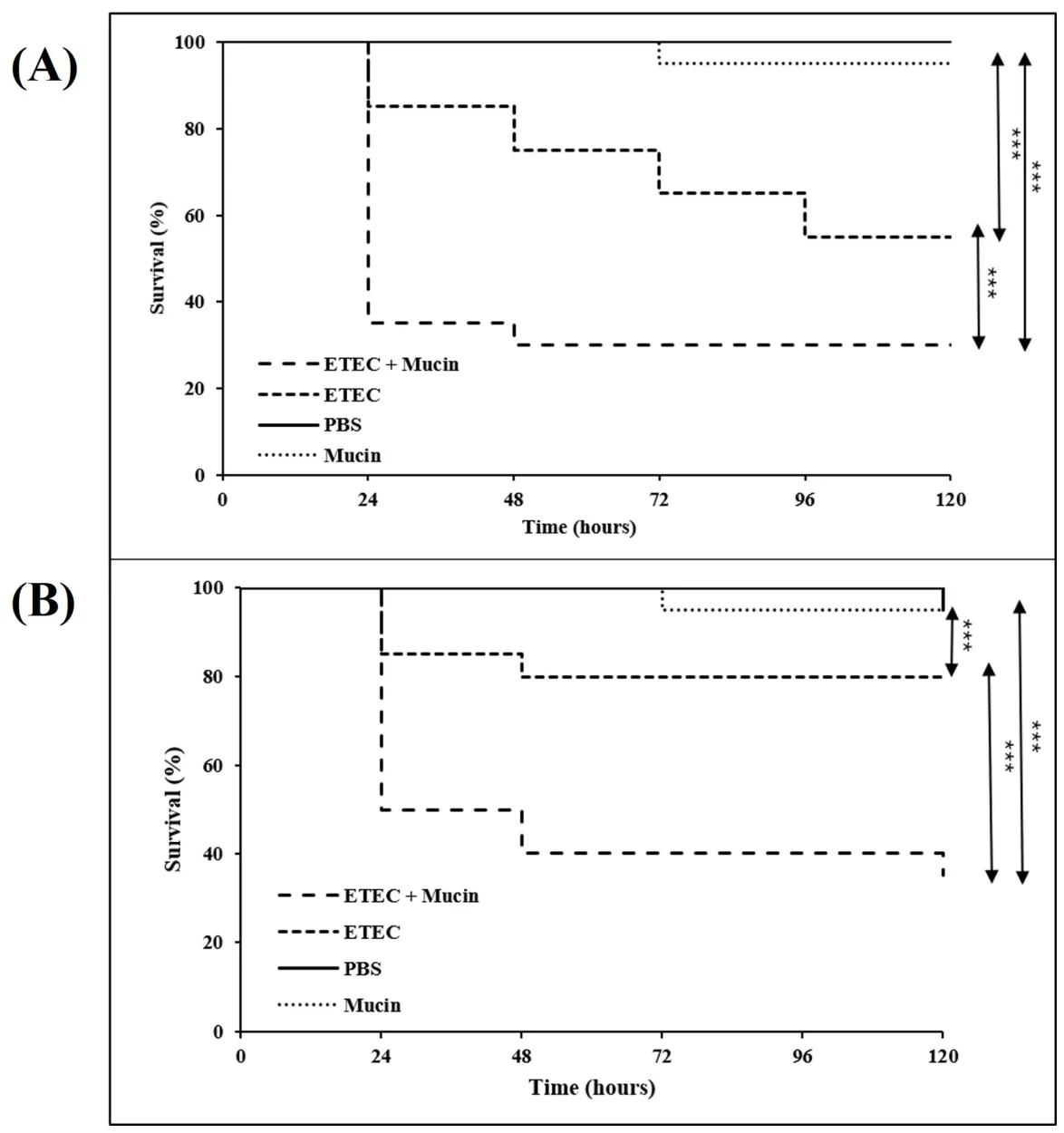
Survival of G. mellonella larvae without and with ETEC, either or not grown in the presence of mucin (1000 mg/L). Larvae were injected with phosphate-buffered saline (PBS), ETEC H10407, or ETEC H10407 cultured and prepared in the presence of 1000 mg/L mucin. Kaplan–Meier survival curves are shown separately for two independent experiments (**A**,**B**), each with 20 larvae per treatment. Asterisks indicate significant differences (log-rank test: *** *p* < 0.001).

Challenge assays using the invertebrate infection model *G. mellonella* were performed to evaluate the effect of mucin exposure on the virulence of ETEC H10407 (Figure 4).

Larvae injected with PBS alone exhibited minimal mortality throughout the 120 h observation period, with 100% and 95% survival at the final time point in two independent experiments, confirming that the injection procedure itself had a negligible effect on larval survival. In contrast, injection with ETEC H10407 significantly decreased larval survival, with 55–80% of the larvae remaining alive after 120 h. Throughout the experiments, larvae infected with mucin-exposed ETEC H10407 consistently exhibited lower survival than those infected with bacteria grown in the absence of mucin.

## 4. Discussion

Mucin is one of the first host-derived components encountered by ETEC upon reaching the intestinal mucosa, making it a potential host cue that may enable the pathogen to adapt to the host environment [5,20]. Despite its central role at the host-microbe interface, little is known about how ETEC responds to host-derived cues such as mucin [1,4]. Here, we demonstrated that physiologically relevant concentrations of mucin modulate multiple virulence-associated traits of ETEC H10407, including swimming motility and biofilm formation. These changes were accompanied by increased virulence in the *G. mellonella* infection model. Collectively, our findings suggest that mucin acts as an important host-derived cue that shapes ETEC behavior and enhances traits associated with colonization and infection, consistent with host-sensing mechanisms described in other enteric pathogens [10,11,21,22,23,24].

Mucin strongly increased swimming motility to such an extent that we had to adapt the assay in order to enable us to perform a statistical analysis, because in the treatments with 250 mg/L mucin or more, the bacteria moved so fast that the plates were completely covered. In the adapted assay, swimming motility increased in a concentration-dependent manner upon mucin exposure, with the highest concentrations tested (500–1000 mg/L) resulting in an approximately twofold increase compared with the mucin-free control. Similar effects of mucin on bacterial motility have been reported for other enteric pathogens, including *Campylobacter jejuni* and *Vibrio cholerae* [10,11]. Mucin-induced motility may facilitate bacterial migration through the intestinal mucus layer, allowing pathogens to reach and colonize the underlying epithelium more efficiently.

Mucin exposure also increased biofilm formation by ETEC H10407 in a concentration-dependent manner, with biofilm biomass reaching more than 4.5-fold higher levels than the mucin-free control at the highest concentration tested. Biofilm formation is considered an important factor contributing to host colonization, as it may enhance bacterial persistence and provide protection against environmental and host-associated stresses [8]. This is consistent with earlier work showing that mucin can promote biofilm formation by environmental strains of *E. coli* [14] under conditions where biofilm would otherwise form poorly. The increase in biofilm formation observed with increasing mucin concentrations was accompanied by enhanced motility and increased mortality of *G. mellonella* larvae, suggesting that mucin may influence multiple virulence-associated phenotypes in ETEC H10407. One limitation of the present study is that the optical density measurements used for bacterial growth (OD_600_) and biofilm formation (OD_570_/OD_600_) cannot distinguish between metabolically active and non-viable cells. Hence, further viability-based assays (such as live-dead staining) would be needed to determine whether mucin affects cell viability or the proportion of viable cells within the biofilm.

Quorum sensing has been implicated in the regulation of motility, biofilm formation, and virulence-associated traits in *E. coli*, including ETEC [25,26]. However, this was not directly investigated in the present study, and therefore its contribution to the effects of mucin observed here remains unclear. Further studies are needed to determine whether quorum sensing pathways indeed contribute to the mucin-induced changes observed here in ETEC. Swimming motility and biofilm formation are often considered to be regulated in opposite directions, resulting in motile and sessile states, respectively [27]. However, this does not necessarily imply that all regulatory pathways should control these phenotypes in opposite directions. In *E. coli*, motility can contribute to surface colonization and in this way increase subsequent biofilm development [28]. In addition to motility and biofilm formation, additional virulence-associated phenotypes might be affected as well. The expression of additional virulence determinants, such as toxins and adhesins, was not assessed in the present study. Future studies examining a broader range of ETEC virulence factors would therefore be valuable to obtain a comprehensive picture of how mucin influences ETEC pathogenicity.

To determine whether the phenotypic changes induced by mucin were accompanied by increased virulence to a host, the virulence of ETEC H10407 was evaluated using the *G. mellonella* invertebrate infection model. Larvae injected with ETEC H10407 showed significantly lower survival than those injected with PBS, confirming the pathogenicity of this strain in the model. ETEC H10407 co-administered with mucin caused significantly higher larval mortality than ETEC administered alone, resulting in an approximately 1.5- to 3.3-fold increase in larval death by the end of the observation period in two independent experiments. Together, these findings suggest that the phenotypic and transcriptional responses induced by mucin are associated with increased pathogenicity in vivo, supporting the biological relevance of the in vitro responses observed in this study. *G. mellonella* has become an increasingly popular alternative to mammalian infection models because it is inexpensive, ethically favorable, and possesses an innate immune system that shares several functional characteristics with that of mammals, including hemocyte-mediated phagocytosis and antimicrobial peptides [29,30]. The model has previously been used to investigate the virulence of diarrheagenic *E. coli* pathotypes, including enteropathogenic *E. coli* [31] and enteroaggregative *E. coli* [32]. Nevertheless, as an invertebrate model, *G. mellonella* lacks adaptive immunity and the differentiated intestinal epithelium of mammalian hosts [29]. Consequently, whether the mucin-associated increase in virulence observed in this study also occurs during infection of humans remains to be determined.

The molecular mechanism underlying the mucin-induced changes in motility and biofilm formation in ETEC remains unclear. Previous studies indicate that the mucus environment can influence physiology and virulence-associated phenotypes in ETEC, including in the prototype strain H10407 used in this study [33]. Mucin or mucin-derived components may therefore act as host-derived cues that alter bacterial regulatory pathways. In other *E. coli* pathotypes, mucin components have been shown to influence the expression of flagellar genes and motility [34], while host-derived signalling molecules can simultaneously affect motility and biofilm formation through regulatory systems such as QseC [35]. In ETEC, quorum sensing-related signalling has also been associated with changes in motility and virulence-associated factors [26]. These findings raise the possibility that mucin may influence multiple virulence-associated phenotypes through regulatory changes. However, whether mucin directly activates such pathways in ETEC remains to be elucidated. The exact part of mucin that is recognized by ETEC also remains unclear. Studies on *Pseudomonas aeruginosa* and EHEC suggest that bacteria can sense either monosaccharides released from mucin or mucin glycans. Indeed, *P. aeruginosa* responds to mucin glycans through the sensor kinase RetS [36], whereas EHEC detects the mucin sugar fucose via the two-component regulatory system FusRK [37]. Further research is needed to elucidate the mechanism underlying mucin sensing in ETEC and to clarify how this host-derived cue regulates the phenotypic changes observed in this study. In addition to prototype ETEC strain H10407, future studies should investigate a broader range of ETEC isolates, including strains of both human and animal origin, to determine whether they respond similarly.

We finally want to mention that the functional properties of mucin following the preparation and sterilization procedure were not compared with those of untreated mucin. Nevertheless, the use of autoclaved porcine gastric mucin has been reported in previous studies [38,39].

## 5. Conclusions

This study demonstrated that mucin is not merely a structural component of the intestinal mucus layer but also acts as a host-derived cue that modulates the virulence-associated behavior of ETEC H10407. Exposure to physiologically relevant concentrations of mucin strongly increased swimming motility and biofilm formation, and these changes in virulence factors were accompanied by increased virulence in the *Galleria mellonella* infection model. Together, these findings support the hypothesis that ETEC senses mucin as a host-derived cue that promotes bacterial adaptation to the host environment, improving our understanding of the host–pathogen interactions that shape ETEC pathogenicity. This research will then support further investigation of antivirulence strategies that interfere with the sensing of mucin rather than targeting bacterial viability in order to control ETEC infections. Such a novel control strategy could consist of the use of analogues of mucin sugars or glycans that block the response to mucin. As such a strategy would not affect the viability of ETEC, there would be a much lower selective pressure for resistance development than for conventional antibiotics.

## Data Availability

Data will be made available upon reasonable request.

## References

[1] Higginson E.E., Sayeed M.A., Pereira Dias J., Shetty V., Ballal M., Srivastava S.K., Willis I., Qadri F., Dougan G., Mutreja A. (2022). Microbiome Profiling of Enterotoxigenic *Escherichia coli* (ETEC) Carriers Highlights Signature Differences between Symptomatic and Asymptomatic Individuals. mBio.

[2] von Mentzer A., Connor T.R., Wieler L.H., Semmler T., Iguchi A., Thomson N.R., Rasko D.A., Joffre E., Corander J., Pickard D. (2014). Identification of Enterotoxigenic *Escherichia coli* (ETEC) Clades with Long-Term Global Distribution. Nat. Genet..

[3] Fleckenstein J.M., Kuhlmann F.M. (2019). Enterotoxigenic *Escherichia coli* Infections. Curr. Infect. Dis. Rep..

[4] Crofts A.A., Giovanetti S.M., Rubin E.J., Poly F.M., Gutiérrez R.L., Talaat K.R., Porter C.K., Riddle M.S., DeNearing B., Brubaker J. (2018). Enterotoxigenic *E. coli* Virulence Gene Regulation in Human Infections. Proc. Natl. Acad. Sci. USA.

[5] Paone P., Cani P.D. (2020). Mucus Barrier, Mucins and Gut Microbiota: The Expected Slimy Partners?. Gut.

[6] Sheng Y.H., Hasnain S.Z. (2022). Mucus and Mucins: The Underappreciated Host Defence System. Front. Cell. Infect. Microbiol..

[7] Derrien M., van Passel M.W.J., van de Bovenkamp J.H.B., Schipper R., de Vos W., Dekker J. (2010). Mucin-Bacterial Interactions in the Human Oral Cavity and Digestive Tract. Gut Microbes.

[8] Sicard J.-F., Le Bihan G., Vogeleer P., Jacques M., Harel J. (2017). Interactions of Intestinal Bacteria with Components of the Intestinal Mucus. Front. Cell. Infect. Microbiol..

[9] Vogt S.L., Peña-Díaz J., Finlay B.B. (2015). Chemical Communication in the Gut: Effects of Microbiota-Generated Metabolites on Gastrointestinal Bacterial Pathogens. Anaerobe.

[10] Tu Q.V., McGuckin M.A., Mendz G.L. (2008). *Campylobacter jejuni* Response to Human Mucin MUC2: Modulation of Colonization and Pathogenicity Determinants. J. Med. Microbiol..

[11] Liu Z., Wang Y., Liu S., Sheng Y., Rueggeberg K.-G., Wang H., Li J., Gu F.X., Zhong Z., Kan B. (2015). *Vibrio cholerae* Represses Polysaccharide Synthesis To Promote Motility in Mucosa. Infect. Immun..

[12] Sun E., Liu S., Hancock R.E.W. (2018). Surfing Motility: A Conserved yet Diverse Adaptation among Motile Bacteria. J. Bacteriol..

[13] Li X., Bossier P., Dierckens K., Laureau S., Defoirdt T. (2015). Impact of Mucin, Bile Salts and Cholesterol on the Virulence of *Vibrio anguillarum* towards Gnotobiotic Sea Bass (*Dicentrarchus labrax*) Larvae. Vet. Microbiol..

[14] Bollinger R.R., Everett M.L., Wahl S.D., Lee Y.-H., Orndorff P.E., Parker W. (2006). Secretory IgA and Mucin-Mediated Biofilm Formation by Environmental Strains of *Escherichia coli*: Role of Type 1 Pili. Mol. Immunol..

[15] Zhang S., Yang Q., Defoirdt T. (2022). Indole Decreases the Virulence of Pathogenic Vibrios Belonging to the Harveyi Clade. J. Appl. Microbiol..

[16] Dehbanipour R., Ghalavand Z. (2022). Anti-Virulence Therapeutic Strategies against Bacterial Infections: Recent Advances. Germs.

[17] Jorjão A.L., Oliveira L.D., Scorzoni L., Figueiredo-Godoi L.M.A., Prata M.C.A., Jorge A.O.C., Junqueira J.C. (2018). From Moths to Caterpillars: Ideal Conditions for *Galleria mellonella* Rearing for in Vivo Microbiological Studies. Virulence.

[18] Jønsson R., Struve C., Jenssen H., Krogfelt K.A. (2017). The Wax Moth Galleria Mellonella as a Novel Model System to Study Enteroaggregative *Escherichia coli* Pathogenesis. Virulence.

[19] Tsai C.J.-Y., Loh J.M.S., Proft T. (2016). Galleria Mellonella Infection Models for the Study of Bacterial Diseases and for Antimicrobial Drug Testing. Virulence.

[20] Johansson M.E.V., Hansson G.C. (2016). Immunological Aspects of Intestinal Mucus and Mucins. Nat. Rev. Immunol..

[21] Barnhart M.M., Lynem J., Chapman M.R. (2006). GlcNAc-6P Levels Modulate the Expression of Curli Fibers by *Escherichia coli*. J. Bacteriol..

[22] Dwivedi R., Nothaft H., Garber J., Xin Kin L., Stahl M., Flint A., Van Vliet A.H.M., Stintzi A., Szymanski C.M. (2016). L-fucose Influences Chemotaxis and Biofilm Formation in *Campylobacter jejuni*. Mol. Microbiol..

[23] Keeney K.M., Finlay B.B. (2013). Microbiology: EHEC Downregulates Virulence in Response to Intestinal Fucose. Curr. Biol..

[24] Le Bihan G., Sicard J.-F., Garneau P., Bernalier-Donadille A., Gobert A.P., Garrivier A., Martin C., Hay A.G., Beaudry F., Harel J. (2017). The NAG Sensor NagC Regulates LEE Gene Expression and Contributes to Gut Colonization by *Escherichia coli* O157: H7. Front. Cell. Infect. Microbiol..

[25] González Barrios A.F., Zuo R., Hashimoto Y., Yang L., Bentley W.E., Wood T.K. (2006). Autoinducer 2 Controls Biofilm Formation in *Escherichia coli* through a Novel Motility Quorum-Sensing Regulator (MqsR, B3022). J. Bacteriol..

[26] Sturbelle R.T., de Avila L.F.d.C., Roos T.B., Borchardt J.L., Dellagostin O.A., Leite F.P.L. (2015). The Role of Quorum Sensing in *Escherichia coli* (ETEC) Virulence Factors. Vet. Microbiol..

[27] Mika F., Hengge R. (2013). Small Regulatory RNAs in the Control of Motility and Biofilm Formation in *E. coli* and *Salmonella*. Int. J. Mol. Sci..

[28] Wood T.K., González Barrios A.F., Herzberg M., Lee J. (2006). Motility Influences Biofilm Architecture in *Escherichia coli*. Appl. Microbiol. Biotechnol..

[29] Cutuli M.A., Petronio Petronio G., Vergalito F., Magnifico I., Pietrangelo L., Venditti N., Di Marco R. (2019). Galleria Mellonella as a Consolidated in Vivo Model Hosts: New Developments in Antibacterial Strategies and Novel Drug Testing. Virulence.

[30] Ménard G., Rouillon A., Cattoir V., Donnio P.-Y. (2021). Galleria Mellonella as a Suitable Model of Bacterial Infection: Past, Present and Future. Front. Cell. Infect. Microbiol..

[31] Chen R.Y., Keddie B.A. (2021). The *Galleria mellonella*-Enteropathogenic *Escherichia coli* Model System: Characterization of Pathogen Virulence and Insect Immune Responses. J. Insect Sci..

[32] Guerrieri C.G., Pereira M.F., Galdino A.C.M., dos Santos A.L.S., Elias W.P., Schuenck R.P., Spano L.C. (2019). Typical and Atypical Enteroaggregative *Escherichia coli* Are Both Virulent in the *Galleria mellonella* Model. Front. Microbiol..

[33] Sauvaitre T., Van Landuyt J., Durif C., Roussel C., Sivignon A., Chalancon S., Uriot O., Van Herreweghen F., Van de Wiele T., Etienne-Mesmin L. (2022). Role of Mucus-Bacteria Interactions in Enterotoxigenic *Escherichia coli* (ETEC) H10407 Virulence and Interplay with Human Microbiome. npj Biofilms Microbiomes.

[34] Kim J.C., Yoon J.W., Kim C.-H., Park M.-S., Cho S.-H. (2012). Repression of Flagella Motility in Enterohemorrhagic *Escherichia coli* O157: H7 by Mucin Components. Biochem. Biophys. Res. Commun..

[35] Yang K., Meng J., Huang Y., Ye L., Li G., Huang J., Chen H. (2014). The Role of the QseC Quorum-Sensing Sensor Kinase in Epinephrine-Enhanced Motility and Biofilm Formation by *Escherichia coli*. Cell Biochem. Biophys..

[36] Wang B.X., Wheeler K.M., Cady K.C., Lehoux S., Cummings R.D., Laub M.T., Ribbeck K. (2021). Mucin Glycans Signal through the Sensor Kinase RetS to Inhibit Virulence-Associated Traits in *Pseudomonas aeruginosa*. Curr. Biol..

[37] Pacheco A.R., Curtis M.M., Ritchie J.M., Munera D., Waldor M.K., Moreira C.G., Sperandio V. (2012). Fucose Sensing Regulates Bacterial Intestinal Colonization. Nature.

[38] Fernandez-Cantos M.V., Babu A.F., Hanhineva K., Kuipers O.P. (2024). Identification of Metabolites Produced by Six Gut Commensal Bacteroidales Strains Using Non-Targeted LC-MS/MS Metabolite Profiling. Microbiol. Res..

[39] Modesto J.L., Pearce V.H., Townsend G.E. (2023). Harnessing Gut Microbes for Glycan Detection and Quantification. Nat. Commun..

